# Forty years of SNOMED: a literature review

**DOI:** 10.1186/1472-6947-8-S1-S2

**Published:** 2008-10-27

**Authors:** Ronald Cornet, Nicolette de Keizer

**Affiliations:** 1 Department of Medical Informatics, Academic Medical Centre, Universiteit van Amsterdam, P.O. Box 22700 1100 DE Amsterdam The Netherlands

## Abstract

**Background:**

Over a period of 40 years, SNOMED has developed from a pathology-specific nomenclature (SNOP) into a logic-based health care terminology. In spite of its long existence and continuous evolvement, it is yet unknown to what extent SNOMED is used in clinical practice, and what benefits were achieved. The aim of this paper is to investigate this by providing an overview of published studies in which a version of SNOMED was studied or applied.

**Methods:**

This paper analyzes the use of SNOMED over time, as reflected in scientific publications, by means of Medline literature search in PubMed. The search included papers from 1966 until June 2006. For each selected paper the following characteristics were classified: version, medical domain, coding moment (during or after the care process), usage, and type of evaluation (e.g., does SNOMED work, does SNOMED help).

**Results:**

250 papers were included in this research. The number of annual publications has increased, as has the number of domains in which SNOMED is being used. Theoretical studies mainly concern comparison of SNOMED to other terminological systems and SNOMED as an illustration of a terminological systems' theory. Few studies are available on the usage of SNOMED in clinical practice, largely involving coding information and retrieval/aggregation based on SNOMED codes.

**Conclusion:**

The clinical application of SNOMED is broadening beyond pathology. The majority of studies concern proving the value of SNOMED in theory. Fewer studies are available on the usage of SNOMED in clinical practice. Literature gives no indication of the use of SNOMED for direct care purposes such as decision support.

## Introduction

Terminological systems (TS) are often mentioned to be an important prerequisite for making electronic medical records a success. Terminological systems provide terms denoting concepts and their relations from a specific domain and can be used to describe information in a structured and standardized way [1].

Well-known examples are MeSH (Medical Subject Headings) [2] for categorizing literature, the ICD family (International Classification of Diseases) [3] for recording (initially) causes of death and (later) diseases, and the family of SNOMED terminological systems [4]. In 1965, the systematized nomenclature of pathology (SNOP) was developed by the College of American Pathologists. In the next forty years several changes on the number of concepts, the covered domains and the underlying representation formalism lead to the currently available Systematized Nomenclature of Medicine, Clinical Terms (SNOMED CT), as is depicted in Table 1. Some countries (e.g. UK, USA, Spain), and some organizations (e.g. UK's National Health Services, ASTM International's Committee E31 on Healthcare Informatics, Federal Drug Administration) have already licensed SNOMED CT, adopting it as the preferred reference terminology. Worldwide, there is an increasing awareness of SNOMED and its development and implementation. This awareness is stimulated by the potential benefits of using SNOMED CT, such as:

**Table 1:** Years of release of major versions of SNOP and SNOMED (Source: ).


1965	SNOP
1974	SNOMED
1979	SNOMED II
1993	SNOMED Version 3.0
1997	LOINC codes integrated into SNOMED
1998	SNOMED Version 3.5
2000	SNOMED RT
2002	SNOMED CT

• enabling a consistent way of indexing, storing, retrieving and aggregating clinical data across specialties and sites of care,

• enabling structuring and computerizing the medical record, thereby reducing the variability in the way data is captured, encoded and used for clinical care of patients and research,

• enabling automated reasoning, e.g. decision support.

Whereas some of these benefits require the formal rigor of SNOMED CT, others can be obtained with earlier versions of SNOMED.

It is yet unknown to what extent terminological systems such as the SNOMED family are used in clinical practice. Furthermore, scientific evidence is lacking that benefits such as the ones mentioned above were achieved in clinical practice.

The aim of this paper is to provide an overview of published studies in which one of the members of the SNOMED family was the main subject of study or plays a secondary role in a study, e.g. to select a patient population for epidemiologic research or to describe an information system in which a SNOMED version was used to code patients. By this overview we will describe the applications and development of the SNOMED family in the medical literature and we will detect the areas in which these applications have proven their value as well as the areas needing future research.

## Methods

A Medline literature search by PubMed was performed to select papers in which SNOMED plays any role. The search period was from 1966 to June 2006. We used ("SNOMED" OR "SNOP") as search query, thereby selecting all papers that have been MeSH-indexed by "SNOMED" and all papers that used "SNOMED" or "SNOP" as a text word in the title or the abstract. Only papers with an English abstract were included. From all papers found, two independent researchers manually excluded papers that were not on the Systematized Nomenclature of Pathology or on the Systematized Nomenclature of Medicine, e.g. when the author's name was Snop. Next, for each selected paper the following characteristics were classified based on the information contained in the abstract:

∘ *Version *(SNOP, SNOMED, SNOMED II, version 3.0/international, SNOMED version 3.5, SNOMED RT, SNOMED CT)

∘ *Medical domain *(anesthesia, cancer, cardiology, gastroenterology, HIV, nephrology, nursing, orthopedics, pathology, primary care, other/unknown/multiple)

∘ *Coding moment *(coding during and/or for the care process, post-coding for research purposes)

∘ *Usage*:

a. SNOMED is the primary object of study (data quality, prove merits for care process or outcome, content coverage, compare to other TS, illustration of a TS theory, standards for electronic medical record, description of implementation)

b. SNOMED is the secondary object of study (used as example, used in a study to retrieve and analyze within epidemiological studies, used in a study on a particular information system to code or classify)

∘ *Type of evaluation*. If SNOMED was evaluated in the study, the aim/research question of evaluation was determined: does it work (technical evaluation); does it help (effect on care), what are the consequences (for the organization); unknown/not applicable.

All papers have been independently analysed by the two authors, different judgement has been discussed and resolved.

## Results

The PubMed search resulted in 318 hits. Among these hits 64 papers were excluded because of lacking an English abstract and 4 papers were excluded because SNOP was used with a different meaning (e.g. a chemical (SnoPPP)2 or the author's name being Snop). The remaining 250 Medline indexed papers on SNOMED have been published in 80 different journals. The AMIA conference proceedings comprised the largest number of papers (n = 71, 28%). Overall, 43% of the papers were published in medical informatics conference proceedings (AMIA, MIE, Medinfo). The other 57% have been published in a wide variety of medical and medical informatics journals. A majority of the medical journals came from the field of pathology.

**Figure 1:**
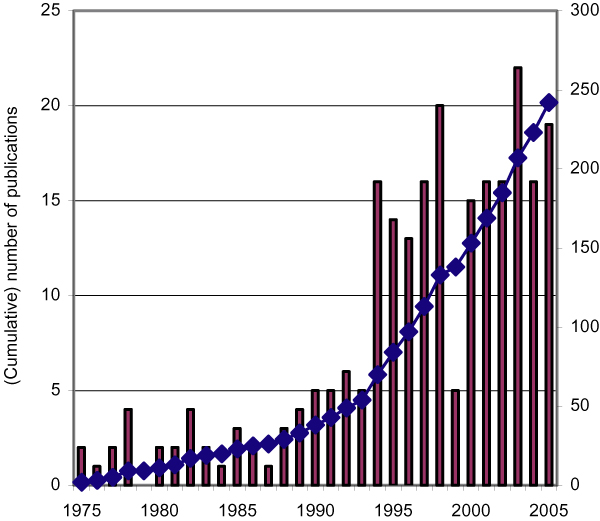
(Cumulative) number of Medline indexed publications on SNOMED from 1975 till 2005.

Figure 1 shows the development of the number of papers on SNOMED in time. The first paper on SNOP having an abstract in PubMed was published in 1975 (NLM began to include abstracts in online citations in 1975). During the early nineties there is a rapid expansion of papers. Since 1994 there is a more or less constant number of 15–20 publications a year.

Review of the 250 included papers on the aspects "version", "medical domain", "coding moment", "usage", and "type of evaluation" provided the following results.

### SNOMED Version

**Figure 2:**
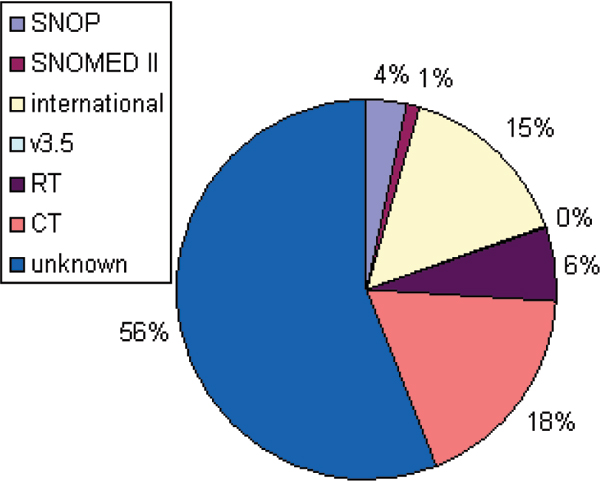
Versions of SNOMED reported in 250 Medline indexed papers (1975–2006).

Half of the papers described the use of "SNOMED" without any further specification of its version. Without specification it could not be determined from the abstracts whether this referred to the 1974 version of SNOMED or any later version. Among the other half, most papers described a study on SNOMED CT (see Figure 2).

### Medical domain

**Figure 3:**
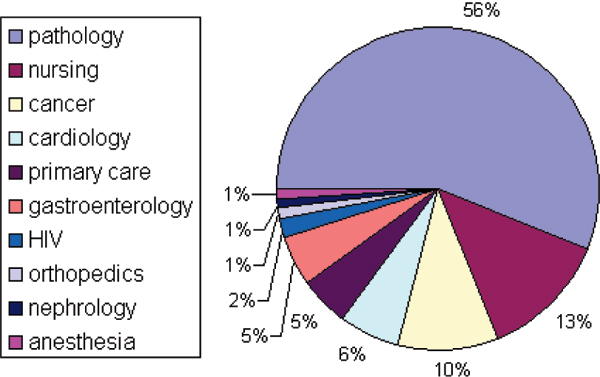
Medical domains of the 100 Medline indexed papers in which a specific medical domain has been described.

Sixty percent (n = 150) of the papers did not clearly describe a specific medical domain of the study or SNOMED was used in the study for multiple disciplines or medicine in general. Among the 40% (n = 100) of the papers in which a specific medical domain had been described, pathology (n = 56), nursing (n = 13) and cancer (n = 10) were most frequently mentioned (see Figure 3).

**Figure 4:**
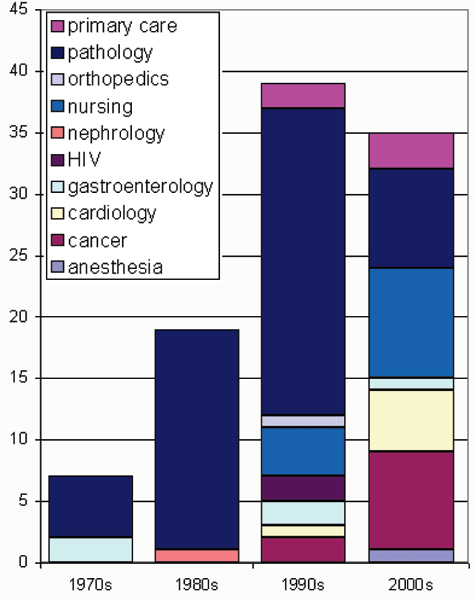
Distribution of medical domains using SNOMED over the years. Note: the 2000s involve the period until June 2006.

Figure 4 shows that in the first 20 years of publications on the SNOMED family almost all papers dealt with pathology. Since 1990 more other medical specialties have started producing studies on SNOMED while since 2000 there has been a minor contribution of pathology among all SNOMED publications.

### Coding moment

For most included papers (n = 150, 60%) it was not clearly described whether concepts were coded using SNOMED during and/or for the purpose of the care process or post-coded for research purposes. In those papers in which this was clearly described, 62% addressed coding that occurred during and/or for the care process, e.g. coding pathology finding in patient reports. In the remainder (38%) coding was performed for research purposes, e.g. post-coding a set of patient cases to compare content coverage of several TSs.

### Usage

**Figure 5:**
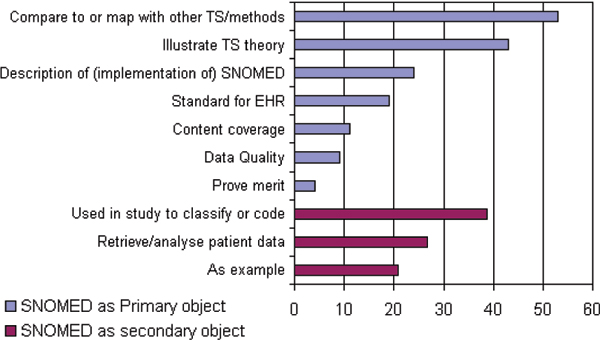
Usage of SNOMED as primary or secondary object of study in 250 Medline indexed papers.

SNOMED was the main object of study in 163 papers. The other 87 papers described a study in which SNOMED plays a secondary role. As is shown in figure 5, there are two major subjects within the studies in which SNOMED is the primary object of study. The first one is the group of studies in which SNOMED is compared to other TSs, mostly on content coverage. The second one is using SNOMED to illustrate a TS theory, e.g. the usefulness of description logics, automatic coding or classification, and/or natural language processing. Among the 87 publications in which SNOMED plays a secondary role, 21 abstracts just mentioned SNOMED as an example of a controlled vocabulary without actually describing its usage, 27 papers described the use of SNOMED codes for retrieval and/or analysis of patient data, and 39 studies described an information system, e.g. an electronic patient record system, in which SNOMED was used to code patient information.

**Figure 6:**
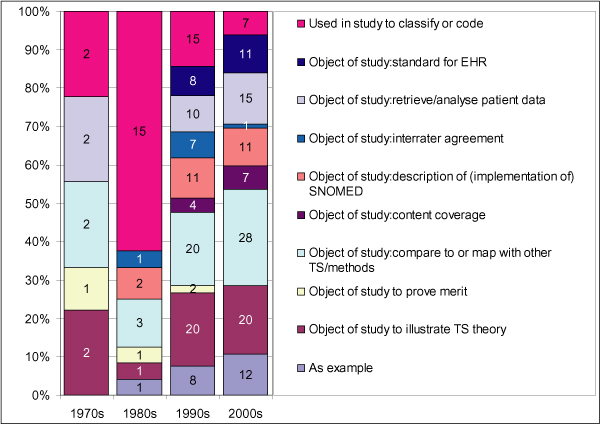
Distribution of SNOMED's usage over the years. Note: the 2000s involve the period until June 2006.

There was no difference in usage among various medical specialties. Figure 6 shows the types of usage of SNOMED over time. Three types of usage occur during the whole period: the use of SNOMED in an information system to classify or code patient information; comparison of a SNOMED version to other TSs such as ICD or MeSH; and SNOMED to illustrate TS theory.

In the earlier years, most studies describe the implementation of a pathology information system in which a SNOMED version is used to code and/or retrieve patients. In the most recent years the usage has become more diverse, with mapping to other systems and illustration of TS theory as the most prominent topics.

Emerging subjects include the use of SNOMED as a standard for the electronic health record and content coverage studies.

### Type of evaluation

52% (n = 129) of the papers described some kind of evaluation of SNOMED. Out of these, 81% (n = 104) concerned a technical evaluation to find an answer to the question "does it work?", e.g. content coverage, automated classification, and 19% (n = 25) evaluated SNOMED on the aspect "does it help?" which mostly concerned the supportive role of SNOMED in retrieving or aggregating patient(group)s and making management reports.

## Conclusion and discussion

This review included 250 papers on members of the SNOMED family. A large number of medical domains and application have been described. Even though in many cases there is no description given of the clinical domain in which SNOMED is being used, the cases in which such a description is given show that the clinical application of SNOMED is broadening beyond pathology.

It is generally known that searching medical bibliographies such as PubMed will not result in finding all published studies on a particular subject [5]. As we searched for the terms SNOP and SNOMED anywhere in the PubMed entry, we are confident that we have captured almost all of the papers in which the SNOMED family of terminological systems plays an important role. However, one must realize that mere use of SNOMED does not necessarily lead to publication. Hence, papers published may well be but the tip of the iceberg, describing cases in which scientific research is being performed on or using SNOMED. About two thirds of the papers described a study in which a SNOMED version was the primary subject of study while the remaining one third concerned studies in which SNOMED plays a secondary role. Publication bias especially will affect our group of papers in which SNOMED plays a secondary role, e.g. for epidemiological studies in which SNOMED codes are used to retrieve eligible patients. The existence of studies in which SNOMED is used to retrieve and select patients is an indication that there are successful implementations of SNOMED in clinical settings although these implementations themselves have probably not been described in the scientific literature.

In many cases it was hard to determine which version of SNOMED was actually used. Mention of the systems was often limited to "SNOMED", not making explicit whether this was the version as released in 1974 or any of its successors. One possible explanation for this is the fact that our study has been based on abstracts and not on full papers. However, checking the full paper of a small sample of abstracts in which the SNOMED version was not specified does not provide more information on the characteristics collected for each paper included.

The two largest groups of papers on a SNOMED version concern the comparison of SNOMED to other TSs and papers in which a theory such as automatic coding, natural language processing and description logics is illustrated by (case studies with) SNOMED. These kinds of studies are particularly relevant for proving the value of SNOMED in theory. Fewer studies are available on the usage of SNOMED in clinical practice. The use of SNOMED as described in these papers largely involves coding information and retrieval/aggregation based on SNOMED codes. Only 2 rather old papers (1975 and 1992) gave some indication of the use of SNOMED to improve the care process or outcome of care. One stated that SNOP provides significant economy in data handling[6]. One should be aware that in 1975 minimizing data storage was much more an economic issue than nowadays. The other paper described how SNOMED could support a quality audit which has discovered substantial deficiencies in performance of a surgical pathology laboratory [7]. Future studies addressing the effect of TS on the care process and outcome are desirable.

Literature gives no indication of the use of SNOMED for direct care purposes such as decision support. As decision support relies on formal representation, which was introduced in SNOMED RT, studies investigating this aspect can now be performed.

## Competing interests

The authors declare that they have no competing interests.

## Authors' contributions

The authors have collaboratively performed the extraction of articles from Pubmed.

Both authors have analyzed the retrieved articles, and independently determined the characteristics.

Both authors have contributed substantially to the manuscript as well as producing the figures.
